# Quantitative phase imaging in common-path cross-referenced holographic microscopy using double-exposure method

**DOI:** 10.1038/s41598-019-46348-9

**Published:** 2019-07-05

**Authors:** Jaromír Běhal

**Affiliations:** Department of Optics, Palacký University, 17. listopadu 1192/12, 771 46 Olomouc, Czech Republic

**Keywords:** Transmission light microscopy, Imaging and sensing

## Abstract

This paper proposes an optimized implementation of the double-exposure method with emphasis on the uniformity and minimization of the residual phase imperfections in cross-referenced holographic microscopy (CRHM). The quantitative phase images are restored from single-shot cross-referenced holograms, which are separated in the Fourier space and processed to eliminate effects caused by imperfections of the optical path and sample background. CRHM is implemented in a microscope configuration supplemented by a Sagnac interference module providing splitting and shearing of the sample and reference waves. Utilization of the averaging process, which enhances precision of quantitative phase image (QPI) reconstruction, applicable in the methods with a replicated field of view is also presented. The high temporal stability of CRHM is verified in calibration measurements and its application potential demonstrated by a quantitative restoration of the phase resolution target and imaging of biological samples including cheek and sperm cells.

## Introduction

Quantitative phase imaging (QPI) is a widely used method for providing information about spatial variations in the optical thickness of weakly absorbing objects that enables to measure the refractive index^1^ and the dry mass density distribution of individual cells^2^. The QPI can be implemented by digital holography which is a label-free technique suitable for observation of living cells. The phase is restored from the interference pattern whose modulation depends on the optical path difference (OPD) of the well-defined reference wave and the signal wave scattered by the specimen. In holographic microscopes using in-line configuration, the temporal stability is guaranteed intrinsically but holographic images overlap with nondiffracted light making it impossible to restore the phase from a single hologram correctly. Multiple holograms must be recorded and the phase-shifting algorithm implemented so that dynamically evolving processes can not be examined by this technique. The single-shot phase reconstruction is possible in an off-axis arrangement, where the holographic image (real or virtual) can be separated by Fourier filtering^3^. Unfortunately, applications of the conventional off-axis arrangement using Mach-Zehnder or Michelson interferometer are limited by loss of temporal stability. The single-shot hologram reconstruction maintaining the high temporal stability is feasible in a common-path setup, in which interfering waves share nearly the same optical path. In self-referencing techniques^4–8^, a part of the beam unscattered by the sample acts as a cross-reference (CR). These techniques are appropriate for imaging the sparse objects with the restriction that only half of the field of view being exploited. In methods with a replicated field of view, one of the beams can be additionally filtered in the Fourier plane to create an isolated reference beam with reduced information about the object^9,10^. This approach enables imaging of the dense samples but requires a precise alignment of added optical components. Both approaches were advantageously implemented using the Sagnac interferometer^11,12^ allowing a continuous adjustment of the angle between the interfering waves. In this publication the optimal use of the double-exposure method often used in the cross-referenced holographic microscopy (CRHM) for aberration elimination is presented. CRHM works in a stable common-path geometry allowing a single-shot quantitative restoration of phase images, while enhancement of the imaging performance is achieved by processing the cross-referenced holograms.

## Methods

**Figure 1:**
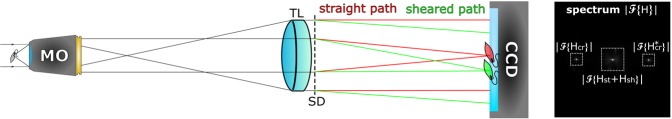
Illustration of straight and sheared optical paths in standard microscope supplemented by a shearing device. MO … microscope objective, TL … tube lens, SD … shearing device, CCD … camera. Typical Fourier amplitude spectrum on the right part of the figure.

In CRHM, the sample and reference waves leaving a conventional microscope configuration are directed into two slightly inclined optical paths, here called straight and sheared optical paths (Fig. 1). Assuming monochromatic coherent illumination with the wavelength *λ* and a lateral shear along the *x*-axis, the hologram captured by the CCD is given by

1 $$H={|{U}_{S}(x)+{U}_{R}(x)+[{U}_{S}(x-{\rm{\Delta }}x)+{U}_{R}(x-{\rm{\Delta }}x)]\exp (-i{k}_{x}x)|}^{2},$$

where *k*_*x*_ = *k* sin *α*, *k* = 2*π*/*λ* and *U*_*S*_(*x*), *U*_*R*_(*x*) and *U*_*S*_(*x* − Δ*x*), *U*_*R*_(*x* − Δ*x*) are the signal and reference waves passing through the straight and sheared optical paths and Δ*x* and *α* define lateral shear of the waves at the CCD and their angular inclination, respectively. The entire hologram (1) consists of two conventional holograms *H*_*st*_ and *H*_*sh*_ created in the straight and sheared paths, respectively, and two complex conjugated cross-referenced holograms, *H*_*cr*_ and ${H}_{cr}^{\ast }$,

2 $$H={H}_{st}+{H}_{sh}+{H}_{cr}\,\exp (\,-\,i{k}_{x}x)+{H}_{cr}^{\ast }\,\exp (i{k}_{x}x),$$

where

$$\begin{array}{rcl}{H}_{st} & = & |{U}_{S}(x)+{U}_{R}(x{)|}^{2},\\ {H}_{sh} & = & |{U}_{S}(x-{\rm{\Delta }}x)+{U}_{R}(x-{\rm{\Delta }}x{)|}^{2},\\ {H}_{cr} & = & [{U}_{S}(x-{\rm{\Delta }}x)+{U}_{R}(x-{\rm{\Delta }}x)][{U}_{S}^{\ast }(x)+{U}_{R}^{\ast }(x\mathrm{)].}\end{array}$$

**Figure 2:**
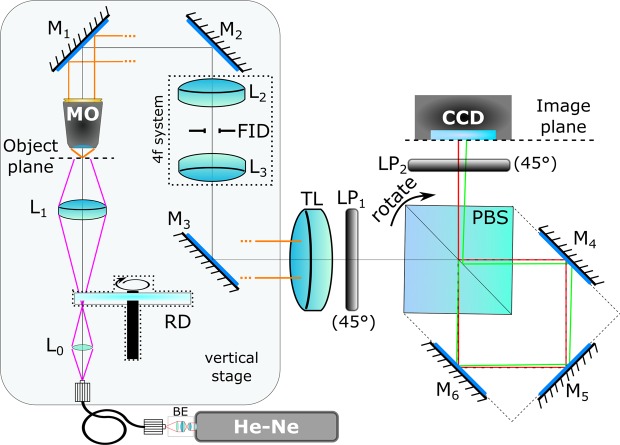
Setup for CRHM using Sagnac interferometer as a shearing device: BE … collimation and focusing lenses, *L*_0_… aspheric lens, RD … rotating diffuser, *L*_1_… condenser lens, MO … microscope objective, *M*_1_ − *M*_4_… mirrors, *L*_2_, *L*_3_… relay lenses, FID… field iris diaphragm, TL … tube lens, *LP*_1_, *LP*_2_… linear polarizers, PBS … polarizing beam splitter, CCD … camera.

Using the demodulation (i.e., filtering in the Fourier domain), the cross-referenced holograms are effectively separated, hence a single-shot phase restoration is in principle possible. Typical Fourier spectrum, including three spatially separated diffraction orders, is presented on the right part of the Fig. 2, where the valuable Fourier terms ${\mathcal F} \{{H}_{cr}\}$ and ${\mathcal F} \{{H}_{cr}^{\ast }\}$ are modulated on the spatial carrier frequencies (2). The cross-referenced hologram *H*_*cr*_ still consists of four interference terms. In the terms of interest, ${U}_{S}(x-{\rm{\Delta }}x){U}_{R}^{\ast }(x)$ and ${U}_{S}^{\ast }(x){U}_{R}(x-{\rm{\Delta }}x)$, the signal wave originating from the sheared and straight path is accessible for processing. The term ${U}_{R}(x-{\rm{\Delta }}x){U}_{R}^{\ast }(x)$ created by the sheared and straight reference wave is used to eliminate the imperfections of the optical path. The interference term ${U}_{S}(x-{\rm{\Delta }}x){U}_{S}^{\ast }(x)$ is insignificant because the sheared and straight signal waves are not spatially overlapped in the observed field of view in real experiments. To demonstrate phase restoration from the recorded holograms the complex amplitude of the signal and reference waves is written using the amplitudes *A*_*S*_(*x*) and *A*_*R*_(*x*) and the phases Φ_*S*_(*x*) and Φ_*R*_(*x*),

3 $${U}_{S}(x)={A}_{S}(x)\,\exp [i{{\rm{\Phi }}}_{S}(x)+ik{W}_{S}(x)],$$

4 $${U}_{R}(x)={A}_{R}(x)\,\exp [i{{\rm{\Phi }}}_{R}(x)+ik{W}_{R}(x)],$$

where *W*_*S*_(*x*) and *W*_*R*_(*x*) denote optical wave aberrations imposed on the signal and reference waves when passing through the optical system. Using well-separated terms of the cross-referenced holograms the signal wave originating from the sheared and straight optical path is reconstructed while eliminating the systematic aberrations of the system,

5 $${{\rm{\Delta }}{\rm{\Phi }}}_{sh}(x-{\rm{\Delta }}x)={\rm{\arg }}\{\frac{{U}_{S}(x-{\rm{\Delta }}x){U}_{R}^{\ast }(x)}{{U}_{R}(x-{\rm{\Delta }}x){U}_{R}^{\ast }(x)}\},$$

6 $${{\rm{\Delta }}{\rm{\Phi }}}_{st}(x)={\rm{\arg }}\{\frac{{U}_{S}^{\ast }(x){U}_{R}(x-{\rm{\Delta }}x)}{{U}_{R}^{\ast }(x){U}_{R}(x-{\rm{\Delta }}x)}\}.$$

The phase restored from (5) and (6) is given by

7 $${{\rm{\Delta }}{\rm{\Phi }}}_{sh}(x-{\rm{\Delta }}x)={\rm{\Delta }}{\rm{\Phi }}(x-{\rm{\Delta }}x)+{\rm{\Delta }}{W}_{SR}(x-{\rm{\Delta }}x),$$

8 $${{\rm{\Delta }}{\rm{\Phi }}}_{st}(x)=-\,{\rm{\Delta }}{\rm{\Phi }}(x)-{\rm{\Delta }}{W}_{SR}(x),$$

where ΔΦ(*x*) = Φ_*S*_(*x*) − Φ_*R*_(*x*) and Δ*W*_*SR*_(*x*) = *W*_*S*_(*x*) − *W*_*R*_(*x*). Since CRHM is implemented in a common-path setup and the signal and reference waves are passing through the same path, optical aberrations degrade both waves equally, hence Δ*W*_*SR*_(*x*) = Δ*W*_*SR*_(*x* − Δ*x*) = 0. In experiments, the term ${U}_{R}(x-{\rm{\Delta }}x){U}_{R}^{\ast }(x)$ used for aberration correction is obtained from a reference hologram taken without sample applying the so-called double-exposure method^13,14^. In the aberration correction, the terms appearing in the numerator and the denominator of Eqs (5) and (6) come from the holograms acquired with and without the sample, respectively. If the phase inaccuracies stay unchanged between capturing both holograms, the aberrations *W*_*S*_(*x*) and *W*_*R*_(*x*) are still mutually eliminated and the terms ΔΦ(*x* − Δ*x*) and ΔΦ(*x*), representing the phase difference between the signal wave and the well-defined reference wave, are restored.

## Results and Discussion

The proposed CRHM was implemented in the setup with built-in Sagnac interferometer working as an adjustable shearing device (Fig. 2). The light beam from unpolarized He-Ne laser (10 mW, *λ* = 633 nm) is transformed using collimation and focusing optics and subsequently spatially filtered by a single-mode fiber. The light emanating from the fiber is captured by an aspheric lens *L*_0_ and focused on a rotating diffuser RD. The light spot created on the RD is scattered toward a lens *L*_1_ illuminating the specimen. Using a microscope objective MO (UPlanFL N, *NA* = 0.75) and a tube lens TL (achromatic doublet, *f* ′ = 400 mm) the magnified image of the specimen (lateral magnification 89×) is created on a CCD (Retiga 4000R, 7.4 *μ*m pixel size, 2048 × 2048 pixels). Relay lenses *L*_2_ and *L*_3_ (both achromatic doublets, *f* ′ = 50 mm) are used to image the field iris diaphragm (FID) to the CCD plane.

The Sagnac interferometer placed between the TL and the CCD consists of a polarizing beam splitter PBS and three mirrors *M*_4_, *M*_5_ and *M*_6_. The polarizers *LP*_1_ and *LP*_2_ placed at the input and output planes of the PBS are oriented at the angle of 45^*o*^ and are used to increase the contrast of interference fringes in the image plane. The lateral shear Δ*x* of the interfering waves is adjusted by slight angular rotation of the PBS. The rotating diffuser allows adjustment of the spatial coherence of illuminating light, reducing speckle noise in the image plane^15,16^ and improves direct optical imaging when the optical system is focused on the specimen.

**Figure 3:**
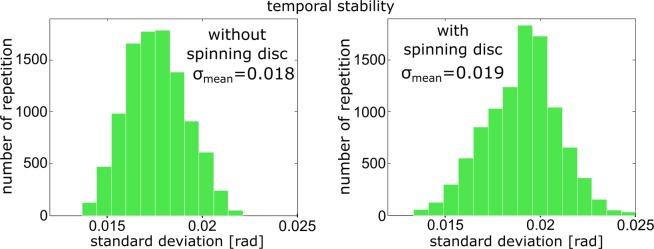
Measurement of the temporal stability carried out with and without rotating diffuser.

The temporal stability of CRHM was tested using 240 holograms recorded in the time interval of 2 minutes. The holograms were taken without specimen and the spatial period of the interference fringes was appropriately adjusted (size of three CCD pixels). The stored holograms then were filtered in the Fourier domain and the phase maps evaluated in the area 8.3 × 8.3 *μ*m^2^. The mean standard deviation is determined as *σ*_*mean*_ = 0.018 rad when the diffuser was out of action. The measurement carried out with the rotating diffuser results in *σ*_*mean*_ = 0.019 rad verifying a low influence of diffuser vibrations on the temporal stability. The results obtained are shown in Fig. 3.

**Figure 4:**
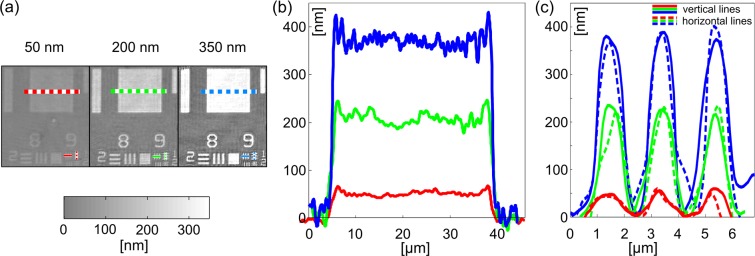
Evaluation of CRHM accuracy by the quantitative phase resolution target USAF 1951. (**a**) Gray level maps with the ground-truth heights 59 nm, 218 nm and 384 nm. (**b**) Cross-sections along thick dashed lines. (**c**) Cross-sections along thin solid and dashed lines passing through the group 9 and element 1 of the USAF target (linewidth 0.98 μm).

The accuracy of CRHM was examined by the quantitative phase resolution target USAF 1951 providing the heights changing in the range from 50 nm to 350 nm with the step 50 nm. The measurement was carried out in three square areas of the target with the ground-truth heights 59 nm, 218 nm and 384 nm (Fig. 4). The phase stroke is determined by comparing the phase restored from a single-shot recording with the phase of the background obtained by the reference hologram. The height is calculated as *h*(*x*) = ΔΦ_*st*_(*x*)/[*k*(*n* − 1)], where the known refractive index of the glass *n* = 1.52 is used for the wavelength *λ* = 633 nm. The phase stroke reconstructed in three parts of the USAF resolution target and converted to the heights is illustrated in Fig. 4(a). Firstly, the standard deviations obtained from the measurement in the square areas of 8.3 × 8.3 *μ*m^2^ using the ground-truth heights 59 nm, 218 nm and 384 nm are determined as 7 nm, 18 nm and 28 nm, respectively. Significant oscillations near the sharp edges of the square phase areas, influencing quality of the phase reconstruction, that are apparent from the cross-sections illustrated in Fig. 4(b) appear as a result of the filtering the high spatial frequencies of the USAF resolution target. Furthermore, cross-sections plotted in the Fig. 4(c) demonstrate that the phase resolution remained preserved even for a group of 0.98 μm width lines of the resolution target.

**Figure 5:**
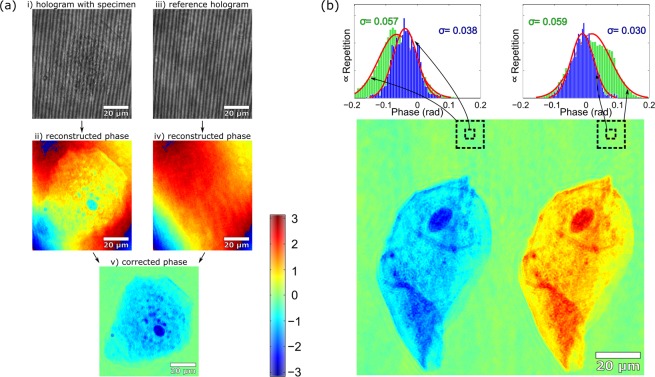
(**a**) Aberration correction using the cross-referenced hologram. (i) Hologram of the cheek cell disturbed by aberrations. (ii) Quantitative phase image of the cell reconstructed from aberrated hologram. (iii) Cross-referenced hologram recorded without the cell (cell relocated by the stage). (iv) Quantitative phase map of the cross-referenced hologram. (v) Aberration corrected quantitative phase image of the cell obtained by subtracting (iv) from (ii). (**b**) Quantitative phase image of the cheek cell with demonstration of nonuniformity of the phase background in the two laterally displaced areas. The standard deviations were increased from 0.038 rad to 0.057 rad and from 0.030 rad to 0.059 rad when evaluation areas expanded from 4.2 × 4.2 *μ*m^2^ (blue line histograms) to 12.5 × 12.5 *μ*m^2^ (green line histograms).

For practical utilization of the method, cheek cells scratched from the inside of a mouth by a toothpick, diffused in a drop of water and sandwiched between ground and cover glass were used as a specimen. The hologram of a cheek cell captured in CRHM setup is shown in Fig. 5(a). The phase image restored from the hologram reveals a strong phase degradation, making the quantitative phase assessment of the image difficult. To correct aberration effects, the specimen was moved out of the field of view and the reference hologram was taken. The phase map restored using the reference hologram is then subtracted from the aberrated phase image. By this operation, phase image of the cell is obtained in which the phase aberrations are eliminated, and the phase background of the image approaches the zero phase level [Fig. 5(a)].

Uniformity of the background phase is evaluated by examining changes of the statistical parameters when enlarging the evaluation region. The results obtained are presented in Fig. 5(b) showing the quantitative double phase image of a cheek cell. The standard deviations evaluated in two small laterally displaced areas of the size 4.2 × 4.2 *μ*m^2^ are determined as 0.030 rad and 0.038 rad, respectively. When the areas are expanded to 12.5 × 12.5 *μ*m^2^, the standard deviations are increased to 0.057 rad and 0.059 rad indicating a nonuniformity of the background phase.

The situation is also documented by blue line and green line histograms related to smaller and larger areas, respectively. In addition to the increased standard deviation, the displacement of the mean value is also evident for the larger evaluation area. The nonuniformity of the background phase demonstrated in Fig. 5(b) is a result of a mechanical relocation of the specimen out of the field of view when capturing the reference hologram. Aberrations occurring in a common optical path are eliminated by the double-exposure method, but phase distortions that differently affect the signal and reference waves are still present. When relocating the specimen using the stage, phase inhomogeneities caused by the ground and cover glass or the surrounding environment have a different effect on the signal and reference waves [*W*_*S*_(*x*) ≠ *W*_*R*_(*x*)]. Hence, the residual phase nonuniformity remains on the phase background even after application of the double-exposure method as demonstrated in Fig. 5(b).

**Figure 6:**
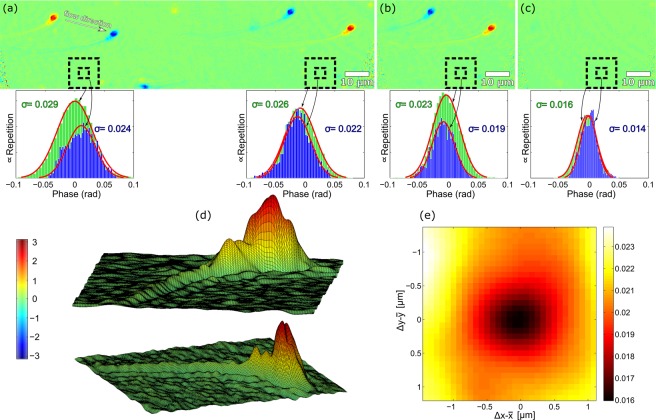
Quantitative phase imaging of the sperm cell. (**a**) Doubled phase image of the cell with demonstration of the background uniformity. The standard deviation is less dependent on the size and lateral position of the evaluation areas than in the previous case (size of areas 4.2 × 4.2 *μ*m^2^ and 12.5 × 12.5 *μ*m^2^). (**b**) The phase image with decreased standard deviation provided by the averaging procedure. (**c**) The phase map of imperfections added by the cross-referenced waves and standard deviations calculated in the marked areas. (**d**) Surface plot of the cell. (**e**) Standard deviation of $[{{\rm{\Delta }}{\rm{\Phi }}}_{st}(x,y)+{{\rm{\Delta }}{\rm{\Phi }}}_{sh}(x-{\rm{\Delta }}x+\bar{x},y-{\rm{\Delta }}y+\bar{y})]/2$ when eliminating the lateral shear Δ*x* in *x*-direction and Δ*y* in *y*-direction.

Elimination of the phase imperfections operates even more efficiently when the reference hologram is recorded without removing the specimen by external mechanical interventions. The situation is demonstrated in the experiment using a semen specimen diluted in water and sandwiched between the ground and cover glass. To achieve moving of the specimen by flowing a surrounding medium, the stage was slightly tilted from the horizontal position. After capturing the specimen hologram, the reference hologram was recorded after the sample was freely displaced outside the field of view. The doubled quantitative phase images of the semen cell restored from the holograms is shown in Fig. 6(a) on the opposite sides of the field of view. The standard deviation evaluated in two laterally displaced areas of the size 4.2 × 4.2 *μ*m^2^ is determined as 0.024 rad and 0.022 rad, respectively. When enlarging the evaluation areas to 12.5 × 12.5 *μ*m^2^, the standard deviation remain almost unchanged providing values of 0.029 rad and 0.026 rad. The improvement of the uniformity of the background phase is also well documented by the blue line and green line histograms demonstrating the measurement in the smaller and larger area, respectively [Fig. 6(a)]. Note the different range of values compared to the Fig. 5(b).

Performance of the phase restoration in CRHM is further enhanced when both straight and sheared images ΔΦ_*st*_(*x*) and ΔΦ_*sh*_(*x* − Δ*x*) are simultaneously available. In the processing of these images, the lateral shear is eliminated numerically and the averaging of the phase differences −ΔΦ_*st*_(*x*) and +ΔΦ_*sh*_(*x*) carried out. Benefits of the averaging procedure are clearly demonstrated in the experiment with the sperm specimen, in which the doubled cell image fully fits into the field of view. Applying the averaging of the phase differences, the image shown in Fig. 6(b) was obtained with the standard deviation of the background reduced to 0.019 rad and 0.023 rad, in the evaluation areas. The surface plot of the restored phase image of the sperm cell and homogeneous background is available in Fig. 6(d).

The terms +ΔΦ_*st*_(*x*) and +ΔΦ_*sh*_(*x* − Δ*x*) contain identical information about the sample [except the sign minus in Eqs (7), (8)] but differ in residual phase imperfections rising from the sheared and the straight cross-reference waves, respectively. The feature mentioned was exploited when numerically eliminating a lateral shear. The sheared image is localized, shifted towards the straight image by $\bar{x}$ and both areas are averaged. The lateral shear is canceled when a standard deviation of the averaged image $[{{\rm{\Delta }}{\rm{\Phi }}}_{st}(x)+{{\rm{\Delta }}{\rm{\Phi }}}_{sh}(x-{\rm{\Delta }}x+\bar{x}\mathrm{)]/2}$ reach its minimum (i.e., in case when $\bar{x}={\rm{\Delta }}x$) because the information about sample is reduced. This property is evaluated in means of a standard deviation calculated inside the larger area 12.5 × 12.5 *μ*m^2^ without the cells [Fig. 6(e)]. The lateral shear is canceled with precision ∝ 0.08 *μ*m which is lower than radius of Airy disc ∝ 0.5 *μ*m in the object space. After removing the lateral shear the phase differences +ΔΦ_*st*_(*x*) and +ΔΦ_*sh*_(*x*) can be averaged which enables to estimate amount of phase fluctuations added into the area of interest by the cross-reference waves, passing through the surrounding medium. Figure 6(c) illustrates the result with the standard deviations of the background 0.014 rad and 0.016 rad which simultaneously estimates the precision of the quantitative phase reconstruction in the evaluation areas. These values converted to OPD result in 1.4 nm and 1.6 nm, respectively. These imperfections can be minimized and CRHM further enhanced in microfluidic applications using channels, where the reference hologram can be captured without mechanical movements and the cross-reference waves pass through the areas out of the channel, without the surrounding medium, which reduce the added phase fluctuations naturally.

## Conclusion

In conclusion, CRHM that works in a temporally stable common-path setup and uses Sagnac interferometer as a shearing device was presented. Interference of sample and reference waves with their sheared replicas was utilized for single-shot holographic reconstruction which was improved by processing procedures for cancelation of the phase defects. The high temporal stability of the setup enabled deploying the double-exposure method in quantitative phase measurements with emphasis on the uniformity and minimization of the residual background phase imperfections. The averaging process applicable in the methods with duplicated field of view for the enhancement of the precision of the phase reconstruction was also presented. The accuracy of the quantitative phase imaging was evaluated in the calibration measurements using the quantitative phase target. Biological samples including cheek and sperm cells were used to demonstrate practical utilization of the method with measurement precision of 1.6 nm for sperm cells.

## Data Availability

The dataset generated or analyzed during the current study are available from corresponding author upon reasonable request.
